# A Retrospective Analysis of the Effect of Combination of Pure Fish Oil with Third Generation Lipid Emulsion on Liver Function in Children on Long-Term Parenteral Nutrition

**DOI:** 10.3390/nu11102495

**Published:** 2019-10-17

**Authors:** Mikołaj Danko, Aleksandra Żyła-Pawlak, Janusz Książyk, Katarzyna Olszewska-Durkacz, Marta Sibilska, Joanna Żydak, Katarzyna Popińska

**Affiliations:** Department of Paediatrics, Nutrition and Metabolic Diseases, The Children’s Memorial Health Institute, Al. Dzieci Polski 20, 04-730 Warsaw, Poland

**Keywords:** fish oil-based lipid emulsion, parenteral nutrition, intestinal failure associated liver disease, children

## Abstract

Background: Deterioration of liver function, or intestinal failure-associated liver disease, is often observed in long-term parenterally fed children. Fish oil-based intravenous lipids have been reported to play a role in the prevention and treatment of intestinal failure associated liver disease. Methods: This retrospective analysis included 40 pediatric patients, (20 male and 20 female), median age 38 months (range 1.5–200 months) on long-term (≥1 month) parenteral nutrition who received the parenteral mixtures containing a combination of a third-generation lipid emulsion and pure fish oil because of laboratory liver function abnormalities. The total dose of fish oil from both emulsions for each patient exceeded 0.5 g/kg/day. Data from visits in an outpatient clinic were retrospectively analyzed using the Wilcoxon test, Mann-Whitney test, and Spearman correlation test. Results: The median time of therapy was 149 days (range 28–418 days). There was a decrease of median total and direct (conjugated) bilirubin concentration from 22.23 µmol/L (range 3.42–243 µmol/L) to 10.26 µmol/L (range 3.42–180.58 µmol/L; *p* < 0.005) and 8.55 (range 1.71–212.04 µmol/L) to 6.84 µmol/L (range 1.71–150.48 µmol/L; *p* < 0.007) respectively. A significant decrease in median alanine aminotransferase, aspartate aminotransferase and gamma-glutamyl transferase was also observed. In 11 patients bilirubin concentrations increased or remained unchanged. When compared to the patients who responded to the combination therapy, the patients who did not respond received parenteral nutrition for a longer time prior to the start of the therapy (51 vs. 30 months; *p* < 0.05). Conclusions: The mixture of an intravenous lipid emulsion containing soybean oil, medium-chain triglycerides, olive oil, and fish oil with the addition of pure fish oil emulsion may be helpful in the treatment of liver complications in children on long-term parenteral nutrition.

## 1. Introduction

Long-term parenteral nutrition is a lifesaving treatment for patients with intestinal failure. In the pediatric population, short bowel syndrome (SBS), gastroschisis, pseudoobstruction syndrome, inflammatory bowel disease and congenital enteropathies, are the most frequent indications for long-term parenteral nutrition [1]. One of the most frequent non-infectious complications of long-term parenteral nutrition in children is intestinal failure-associated liver disease (IFALD) [2,3]. The diagnosis of IFALD is usually based on biochemical tests: direct bilirubin greater than 34.2 µmol/L with or without abnormally high liver enzymes for more than two weeks when other causes of cholestasis have been excluded [2,3,4].

IFALD is a severe condition which can lead to liver cirrhosis and liver failure. Risk factors of IFALD include prematurity, low and very low birth weight, necrotizing enterocolitis, intestinal atresia, gastroschisis, omphalocele, diaphragmatic hernia, intestinal motility disorders duration of parenteral nutrition and catheter-related blood-stream infections (CRBI) [4,5,6,7,8].

The etiology of IFALD is still unknown. The content of parenteral admixtures like lipid emulsion, trace elements (manganese, copper) and the dose of amino acids influence liver function. Excessive parenteral energy load may also lead to liver steatosis. The use of soybean oil-based lipid emulsions (SOLE) affect liver function probably because of the large amount of omega-6 polyunsaturated fatty acids (PUFA) which are precursors of pro-inflammatory cytokines and significant content of phytosterols (which have been proven to interfere with farsenoid X receptor signaling in hepatocytes and decrease bile acids transport) [9,10,11,12]. Moreover, recurrent CRBI and circulating bacterial toxins stimulate Kupffer cells to release proinflammatory cytokines, which thereby decrease the liver function. The immaturity of enterohepatic circulation and the accumulation of bile salts in the liver of neonates and infants make IFALD more frequent in children than in adults. A predisposition towards small intestine bacterial overgrowth syndrome (SIBOS) and lack of or limited enteral feeding in children with SBS after intestinal surgery cause absorption of bacterial lipopolysaccharides and toxins from the gut lumen. This occurs because the impaired integrity of intestinal mucosa may affect hepatocytes and stimulate Kupffer cells as well [13,14,15]. Recent research shows that fibroblast growth factor 19 in the liver protects against bile salt accumulation. This growth factor is produced in ileum and its serum concentration in children with SBS was negatively correlated with the grade of portal inflammation and liver fibrosis stage in a study performed by Mutanen et al. [16,17]. Some studies have shown the possible role of gene polymorphisms in the protection from IFALD [18].

Several methods of treatment and prevention of IFALD are mentioned in the literature but none of them has been proven to be effective more than others. Cyclic parenteral nutrition, suspension of lipid administration, prevention of catheter-related bloodstream infections, decontamination of the gastrointestinal tract, administration of cholecystokinin or erythromycin and trophic enteral nutrition are supposed to be helpful for parenterally fed patients with the liver disease. [4,7,19,20,21] The lack of well-conducted randomized clinical trials do not allow us to conclude firm recommendations for the treatment of these patients. In the last 14 years, there were many publications concerning fish oil in the prevention and treatment of IFALD [22,23,24,25,26,27,28,29,30]. It seems that the lack of phytosterols, a high amount of antioxidative alpha-tocopherol and anti-inflammatory omega-3 fatty acids may reduce the risk of liver damage and improve liver function [31,32,33,34,35,36,37]. Although the results of previous studies are promising, further clinical trials are needed [38,39,40]. There are currently three lipid emulsions which contain fish oil (Table 1).

The dose of fish oil used in the treatment of IFALD in children is generally 1.0 g/kg/day [23,24,25,26,27]. This amount was enough to prevent the essential fatty acids deficiency [24], but it may be insufficient to deliver full energy load, especially in neonates, infants, and young children.

In 2013, we combined a third-generation lipid emulsion with pure fish oil in parenteral admixtures to treat liver function disorders in patients on long-term parenteral nutrition. In this paper, we would like to summarize the efficacy of such a treatment.

## 2. Materials and Methods

From 2013 to 2016, we identified 40 pediatric patients, 20 male and 20 female, with a median age of 38 months (range 1.5–200 months) on long-term (≥ 1 month) parenteral nutrition who received a combination of third-generation lipid emulsion and pure fish oil because of abnormally high bilirubin concentrations and/or liver enzymes according to norms in our laboratory (Table 2).

Abnormalities in any of these parameters were sufficient to introduce the therapy. Most patients were part of a parenteral nutrition program with total parenteral nutrition being infused median 7 days/week (range 4–7 days/week). Each patient received a combination of 2 lipid emulsions in parenteral admixture—a third-generation lipid emulsion (20% SmofLipid) and fish oil, with pure fish oil (10% Omegaven). All patients remained parenteral nutrition-dependent during the study. Normalization of liver parameters was the rationale for stopping the therapy with lipids combination.

We analyzed laboratory tests on the introduction of therapy and after at least 28 days of treatment: total and conjugated bilirubin level, the activity of asparagine transaminase (AST), alanine transaminase (ALT), gamma-glutamyl transferase (GGT) and International Normalized Ratio (INR). The changes of patients’ body weight, parenteral energy load, use of ursodeoxycholic acid, the presence of Bauchin’s valve, length of remnant intestine and infectious complications during the therapy were also taken into consideration. Initial aspartate aminotransferase/platelet ratio index (APRI) and its possible influence on treatment response were assessed as well. The data was statistically analyzed using the Wilcoxon test, Mann-Whitney test and Spearman rank correlation test with StatSoft, Inc. (2005, Palo Alto, CA, USA). STATISTICA (data analysis software system), version 7.1. www.statsoft.com.

The research protocol was approved by the Memorial Children’s Health Institute Ethics Committee.

## 3. Results

The main indication for parenteral nutrition in our patients was SBS along with total aganglionosis, chronic intestinal pseudoobstruction syndrome and congenital sodium diarrhea. Among patients with SBS, 13 had remnant small intestine length ≤ 10 cm (ultra-short bowel syndrome) (Table 3).

The therapy with a combination of lipid emulsions lasted a median 149 days (range 28–418 days). The dose of fish oil from both emulsions exceeded 0.5 g/kg/day (median at the beginning of therapy 0.725 g/kg/d, (range 0.57–1.25 g/kg/d). The median dose of third-generation lipid emulsion was 1 g/kg/d (range 0.5–2.0 g/kg/d). The majority of patients had been given the third-generation lipid emulsion before (32 patients). Eight patients did not receive nutrition via the gastrointestinal tract (5 patients with SBS, 2 with aganglionosis and one with pseudoobstruction syndrome). There were two deaths after the end of the study (both related to liver failure, including one after liver transplantation).

The median supply of energy from parenteral nutrition was 67.7 kcal/kg/d (range 37.8–105.0 kcal/kg/d). The median total energy supply (parenteral and enteral) was 82.8 kcal/kg/d (range 44.6–139.9 kcal/kg/d). The median amount of fish and soy oil at the time of inclusion was 0.73 g/kg/d (range 0.58–1.26 g/kg/d) and 0.30 g/kg/d (range 0.15–0.60 g/kg/d) respectively.

There were statistically significant differences in the supply of fish oil from both emulsions at the end of observation of patients who received or did not receive enteral nutrition (*p* < 0.01). Median values were: 0.72 g/kg/d (range 0.54–1.23 g/kg/d) and 1.12 g/kg/d (range 0.73–1.30 g/kg/d) respectively.

There were statistically significant differences in the supply of soy oil from the third-generation lipid emulsion at the end of observation of patients who received or did not receive enteral nutrition (*p* < 0.03). Median values were: 0.30 g/kg/d (range 0.15–0.45 g/kg/d) and 0.45 g/kg/d (range 0.23–0.60 g/kg/d) respectively.

There was a general improvement of liver laboratory tests in patients undergoing treatment. The median total bilirubin level before the introduction of therapy was 22.3 µmol/L (range 3.42–243 µmol/L) and at the end of the therapy decreased to 10.26 µmol/L (range 3.42–180.58 µmol/L), *p* < 0.005. Differences in total bilirubin before introducing the therapy in patients receiving enteral and not receiving enteral nutrition were statistically significant (*p* < 0.04). Median and ranges respectively: 17.1 µmol/L (3.6–239.50 µmol/L) and 61.22 µmol/L (9.23–110.57 µmol/L). Differences in total bilirubin in patients receiving enteral and not receiving enteral nutrition were not statistically significant at the end of the observation.

**Figure 1:**
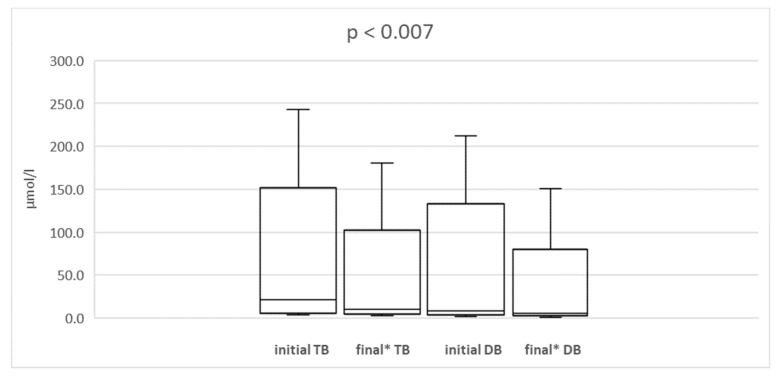
The comparison of initial and final (* at the end of combination therapy) total (TB) and conjugated bilirubin (CB) concentration in the whole group of patients. The lower and upper borders of the box indicate 25th and 75th percentiles, respectively. The line within the box depicts the median. The error bars indicate minimum and maximum.

Median conjugated bilirubin also decreased from 8.55 µmol/L (range 1.71 to 212.04 µmol/L) to 6.84 µmol/L (range 1.71–150.48 µmol/L; *p* < 0.007) (Figure 1).

Differences in conjugated bilirubin before introducing therapy in patients receiving enteral and not receiving enteral nutrition were not statistically significant: median 7.35 µmol/L (range 2.4–212.38 µmol/L) and 52.50 µmol/L (range 3.42–110.12 µmol/L) respectively. The differences reached statistical significance (*p* < 0.04) at the end of the observation. The median concentration of conjugated bilirubin in the group receiving enteral nutrition was 5.30 µmol/L (range 1.71–150–65 µmol/L) and in the group not enterally fed 18.7 µmol/L (range 2.39–101.40 µmol/L).

**Figure 2:**
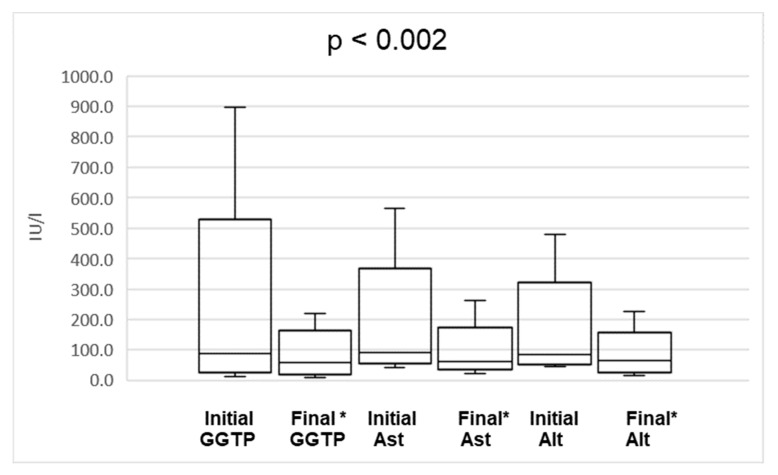
The comparison of initial and final (* at the end of combination therapy) liver enzymes activity. The lower and upper borders of the box indicate 25th and 75th percentiles, respectively. The line within the box depicts the median. The error bars indicate minimum and maximum.

We also observed decrease of median activity of AST from 90 IU/L (range 43–566 IU/L) to 61 IU/L (range 22–262 IU/L; *p* < 0.002), ALT from 85,5 IU/L (range 44–479 IU/L) to 66 IU/L (range 16–226 IU/L; *p* < 0.002) and GGT from 89 IU/L (range 11–897 IU/L) to 58.5 IU/L (range 9–218 IU/L; *p* < 0.002) (Figure 2).

**Figure 3:**
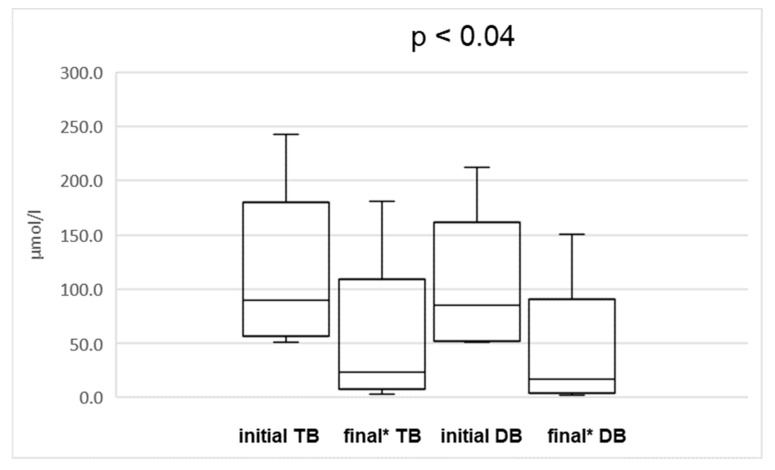
The comparison of initial and final (* at the end of combination therapy) total (TB) and conjugated bilirubin (CB) concentration in initially cholestatic patients. The lower and upper borders of the box indicate 25th and 75th percentiles, respectively. The line within the box depicts the median. The error bars indicate minimum and maximum.

We didn’t observe statistically significant changes in platelet count and INR level during the therapy. The body-weight of patients increased significantly during the treatment, by a median of 11.2%. Out of the whole group, 11 patients did not respond to the therapy—their bilirubin level increased or remained elevated but none of those with initial conjugated bilirubin below 34.2 µmol/L became cholestatic. The median time of parenteral nutrition before the study had been significantly longer in the group of non-responders: 51 vs. 30 months in the whole group (*p* < 0.05). Among these patients, in five of them, the indication for parenteral nutrition was short bowel syndrome, five suffered from intestinal motility disorders (chronic intestinal pseudoobstruction syndrome and total aganglionosis), and one patient had short bowel syndrome combined with cystic fibrosis. In the patients with cholestasis-initial conjugated bilirubin over 34.2 µmol/L (13 out of 40) we observed a significant decrease of all parameters: median total bilirubin from 90.63 µmol/L (range 51.13 to 243 µmol/L) to 23.9 µmol/L (range 3.42 to 181.26 µmol/L; *p* < 0.04), median conjugated bilirubin from 85.5 µmol/L (range 51.3 to 212.04 µmol/L) to 17.1 µmol/L (range 1.71 to 150.48 µmol/L; *p* < 0.02) (Figure 3), median activity of AST from 168 IU/L (range 64 to 380) to 64 IU/L (range 37 to 182 IU/L; *p* < 0.002), ALT from 149 IU/L (range 44 to 226) to 64 IU/L (range 26 to 226 IU/L; *p* < 0.03) and GGT from 214 IU/L (range 87 to 897 IU/L) to 104 IU/L (range 15 to 218 IU/L; *p* < 0.008) (Figure 3).

In the case of patients with intestinal motility disorders (9 out of 40), we observed the worst response to administered treatment. In this group median total and conjugated bilirubin increased slightly during the study, whereas the median activity of liver enzymes decreased–AST from 96 to 67 IU/L; ALT from 83 to 71 IU/L.

During the studied period of time, 8 patients had catheter-related bloodstream infections. At the end of the treatment, we observed a higher median direct bilirubin concentration and GGT activity in these patients in comparison to the whole group (24.62 vs. 11.97 µmol/L and 134 vs. 50 IU/L). The Mann-Whitney test showed that neither the length of remnant bowel nor presence of ileocecal valve and ursodeoxycholic acid administration influenced bilirubin concentration and liver enzymes activity.

In patients with a higher dose of glucose or amino acids in parenteral nutrition mixtures (over 12 g/kg/day and over 2 g/kg/day respectively) at the onset of the therapy, we observed a higher initial serum concentration of direct and total bilirubin and GGT activity (*p* < 0.05).

In the group of prematurely born children (22 out of 40 patients) initial bilirubin concentration, AST and GGT activity were higher than in the whole group (*p* < 0.05).

The initial median APRI was 1.14 (range 0.2–13.8) in the whole group of patients. It was significantly higher in patients with direct bilirubin >34.2 µmol/L or ALT >100 IU/L (median 1.72 range 0.3–13.8) but in the group of non-responders, the median APRI value was 0.91 (range 0.3–3.6). The Spearman rank correlation test showed a positive correlation between conjugated bilirubin concentration and APRI value in patients with conjugated bilirubin >34.2 µmol/L (*p* < 0.05, r = 0.86803).

## 4. Discussion

In 2005, a case report concerning the reduction of IFALD upon introduction of fish oil-based lipid emulsion was published. The case presented a 16-year-old, allergic to soybean oil, who was parenterally fed at home [22]. The information from Boston which followed, concerned larger groups of patients [23,24,25,26,27]. In the USA soy-bean lipid emulsion is used in children’s parenteral nutrition, which is considered a risk factor for IFALD due to the high content of phytosterols and long-chain n-6 fatty acids. Hence, the improvement of the liver function observed in the US studies after changing from 20% soy-bean lipid emulsion to pure fish oil may be clearer than in the case of comparison of fish oil with other, less hepatotoxic, lipid emulsions. The lower dose of lipids used in the case of pure fish oil (maximum 1g/kg/day) could be another reason for such results. Several years ago, some reports referring to admixing of fish oil-based lipids with another lipid emulsion in order to reduce cholestasis were published [41,42]. Combined lipid emulsions containing fish oil as one of their ingredients have been commercially available (20% SmofLipid and 20% Lipidem) for the past 15 years [27,28].

To the best of our knowledge, the present analysis is the first description of patients on long-term parenteral nutrition treated by adding pure fish oil-based lipid emulsion to combined lipid emulsion to increase n-3 fatty acids in parenteral admixture and reverse IFALD.

The results of the present analysis show that the effect of an increased dose of n-3 fatty acids on liver function probably did not depend only on the total lipid dose. The worst response to the therapy observed in patients fed parenterally for a longer period is probably associated with more advanced histopathological changes in the liver tissue. Also, in the study presented by Nandivada, older age of patients at the beginning of the treatment was one of the risk factors for failure of pure fish oil therapy [43]. Patients with intestinal motility disorders, like chronic intestinal pseudoobstruction syndrome, are more vulnerable to bacterial overgrowth and enterotoxaemia that affect the liver function, which may be responsible for the lower effect of intravenous lipids modification [44]. Although the results of the current study are promising, we did not assess the histopathological changes in liver biopsies. Some studies demonstrate that although the laboratory parameters of cholestasis improved in children with IFALD after introducing fish oil therapy, the histological changes in the liver either remain the same or progress [45,46]. On the other hand, Nandivada and Khan demonstrated in their studies that after successful pure fish oil therapy to treat IFALD, serum liver parameters remained low even if patients switched to 20% soybean-based lipid emulsion again [47,48]. The study of Wang demonstrates the opposite results. [49].

Our analysis confirms that prematurity and CRBIs are associated with a higher risk of cholestasis in parenterally fed children. Interestingly, APRI was positively correlated with IFALD but did not correlate with lack of response to the therapy. That might suggest that liver fibrosis in children with IFALD doesn’t predict faster end-stage liver insufficiency [50].

The majority of patients in our study were involved in home parenteral nutrition program so we did not include children with the rapid progress of IFALD which were not stable enough to be discharged from hospital. In our analysis, we have assessed retrospectively a very diversified cohort of pediatric patients without any control group, which is the major limitation of this study.

## 5. Conclusions

Modification of lipid emulsion by adding pure fish oil to combined lipid emulsion may positively influence the liver parameters in children with IF on long-term parenteral nutrition. In our opinion, the described approach could be the “first step” of treatment of liver complications of intestinal failure in infants and children < the age of 2, in whom the maintenance of calories intake may be the most important.

## Figures and Tables

**Table 1:** The characteristics of lipid emulsions containing fish oil.

Lipid Emulsion	10% Omegaven (Fresenius Kabi^®®^)	20% SmofLipid (Fresenius Kabi^®®^)	20% Lipidem (BBraun^®®^)
Soybean oil	-	30%	40%
Olive oil	-	25%	-
Medium chain triglycerides	-	30%	50%
Fish Oil	100%	15%	10%

**Table 2:** Norms used in our Laboratory.

AST IU/L	ALT IU/L	GGT IU/L
6 d.o.–6 m.o.: <84 7–12 m.o: <89 1–3 y.o.: <56 4–12 y.o.: <52 13–17 y.o.: <33	6 d.o–6 m.o.: <60 7–12 m.o: <57 1–12 y.o.: <39 13–17 y.o.: <26	<1 y.o.: <203 1–3 y.o.: <87 4–6 y.o.: <26 7–12 y.o: <31 13–17 y.o.: <29

**Table 3:** The characteristics of studied group of patients at the onset of therapy.

Number of Patients:	40
Sex:	
Male:	20
Female:	20
Median age (range):	38 m.o. (1.5–200)
Median time of PN before the treatment (range)	30.5 months (0.5–166)
Median body weight (range):	13.33 kg (2–40.3)
Diagnosis:	
SBS	30
Motility disorders	9
Others	1
Median days of PN/week (range):	7 (4–7)

## References

[1] Duggan P.C., Jaksic T. (2017). Pediatric Intestinal Failure. N. Engl. J. Med..

[2] Courtney C.M., Warner B.W. (2017). Pediatric intestinal failure-associated liver disease. Curr. Opin. Pediatr..

[3] Al-Shahwani N.H., Sigalet D.L. (2017). Pathophysiology, prevention, treatment, and outcomes of intestinal failure-associated liver disease. Pediatr. Surg. Int..

[4] Lacaille F., Gupte G. (2015). Intestinal failure-associated liver disease: A position paper of the ESPGHAN Working Group of Intestinal Failure and Intestinal Transplantation. J. Pediatr. Gastroenterol. Nutr..

[5] Cavicchi M., Beau P. (2000). Prevalence of liver disease and contributing factors in patients receiving home parenteral nutrition for permanent intestinal failure. Ann. Intern. Med..

[6] Lauriti G., Zani A. (2014). Incidence, prevention, and treatment of parenteral nutrition-associated cholestasis and intestinal failure-associated liver disease in infants and children: A systematic review. J. Parenter. Enteral. Nutr..

[7] Wales P.W., Allen N., Worthington P., George D., Compher C. (2014). Clinical guidelines: Support of pediatric patients with intestinal failure at risk of parenteral nutrition-associated liver disease. J. Parenter. Enter. Nutr..

[8] Goulet O., Joly F. (2009). Some new insights in intestinal failure-associated liver disease. Curr. Opin. Organ. Transplant..

[9] Lee W.S., Sokol R.J. (2015). Intestinal Microbiota, Lipids and the Pathogenesis of Intestinal Failure-Associated Liver Disease. J. Pediatr..

[10] Kurvinen A., Nissinen M.J. (2011). Effects of long-term parenteral nutrition on serum lipids, plant sterols, cholesterol metabolism, and liver histology in pediatric intestinal failure. J. Pediatr. Gastroenterol. Nutr..

[11] Hukkinen M., Mutanen A. (2017). Parenteral Plant Sterols Accumulate in the Liver Reflecting Their Increased Serum Levels and Portal Inflammation in Children with Intestinal Failure. J. Parenter. Enter. Nutr..

[12] Mutanen A., Nissinen M.J. (2014). Serum plant sterols, cholestanol, and cholesterol precursors associate with histological liver injury in pediatric onset intestinal failure. Am. J. Clin Nutr..

[13] Orso G., Mandato C. (2016). Pediatric parenteral nutrition-associated liver disease and cholestasis: Novel advances in pathomechanisms-based prevention and treatment. Dig. Liver Dis..

[14] Pereira-Fantini P.M., Lapthorne S., Altered F.X.R. (2014). Signalling is associated with bile acid dysmetabolism in short bowel syndrome-associated liver disease. J. Hepatol..

[15] Xiao Y.-T., Cao Y. (2016). Altered systemic bile acid homeostasis contributes to liver disease in pediatric patients with intestinal failure. Sci. Rep..

[16] Mutanen A., Lohi J. (2015). Loss of ileum decreases serum fibroblast growth factor 19 in relation to liver inflammation and fibrosis in pediatric onset intestinal failure. J. Hepatol..

[17] van Erpecum K.J., Schaap F.G. (2015). Intestinal failure to produce FGF19: A culprit in intestinal failure-associated liver disease?. J. Hepatol..

[18] Burghardt K.M., Avinashi V. (2014). A CARD9 Polymorphism Is Associated with Decreased Likelihood of Persistent Conjugated Hyperbilirubinemia in Intestinal Failure. PLoS ONE.

[19] González-Contreras J., Villalobos Gámez J.L. (2012). Cholestasis induced by total parenteral nutrition; effects of the addition of Taurine (Tauramin^®®^) on hepatic function parameters; possible synergistic action of structured lipids (SMOFlipid^®®^). Nutr. Hosp..

[20] Raphael B.P., Duggan C. (2012). Prevention and Treatment of Intestinal Failure-Associated Liver Disease in Children. Semin. Liver Dis..

[21] Cober M.P., Teitelbaum D.H. (2010). Prevention of parenteral nutrition-associated liver disease: Lipid minimization. Curr. Opin. Organ. Transplant..

[22] Gura K.M., Parsons S.K. (2005). Use of a fish oil-based lipid emulsion to treat essential fatty acid deficiency in a soy allergic patient receiving parenteral nutrition. Clin. Nutr..

[23] Gura K.M., Duggan C.P. (2006). Reversal of parenteral nutrition-associated liver disease in two infants with short bowel syndrome using parenteral fish oil: Implications for future management. Pediatrics.

[24] Nandivada P., Fell G.L. (2017). Long-Term Fish Oil Lipid Emulsion Use in Children with Intestinal Failure–Associated Liver Disease. J. Parenter. Enteral. Nutr..

[25] Gura K.M., Lee S. (2008). Safety and efficacy of a fish-oil-based fat emulsion in the treatment of parenteral nutrition-associated liver disease. Pediatrics.

[26] de Meijer V.E., Gura K.M. (2010). Parenteral fish oil monotherapy in the management of patients with parenteral nutrition-associated liver disease. Arch. Surg..

[27] Nandivada P., Fell G.L. (2016). Lipid emulsions in the treatment and prevention of parenteral nutrition-associated liver disease in infants and children. Am. J. Clin. Nutr..

[28] Nandivada P., Cowan E. (2013). Mechanisms for the effects of fish oil lipid emulsions in the management of parenteral nutrition-associated liver disease. Prostaglandins Leukot. Essent. Fatty Acids.

[29] Puder M., Valim C. (2009). Parenteral fish oil improves outcomes in patients with parenteral nutrition associated liver injury. Ann. Surg..

[30] Premkumar M.H., Carter B.A. (2013). High rates of resolution of cholestasis in parenteral nutrition-associated liver disease with fish oil-based lipid emulsion monotherapy. J. Pediatr..

[31] Tillman E.M., Helms R.A. (2011). ω-3 long chain polyunsaturated Fatty acids for treatment of parenteral nutrition-associated liver disease: A review of the literature. J. Pediatr. Pharmacol. Ther..

[32] Fallon E.M., Le H.D. (2010). Prevention of parenteral nutrition-associated liver disease: Role of v-3 fish oil. Curr. Opin. Organ. Transplant..

[33] Deshpande G., Simmer K. (2014). Fish Oil (SMOFlipid) and olive oil lipid (Clinoleic) in very preterm neonates. J. Pediatr. Gastroenterol. Nutr..

[34] Lee S., Park H.J. (2016). Reversal of Intestinal Failure-Associated Liver Disease by Switching from a Combination Lipid Emulsion Containing Fish Oil to Fish Oil Monotherapy. J. Parenter. Enter. Nutr..

[35] Tomsits E., Pataki M. (2010). Safety and Efficacy of a Lipid Emulsion Containing a Mixture of Soybean Oil, Medium-chain Triglycerides, Olive Oil, and Fish Oil: A Randomised, Double-blind Clinical Trial in Premature Infants Requiring Parenteral Nutrition. J. Pediatr. Gastroenterol. Nutr..

[36] Goulet O., Antébi H. (2010). A new intravenous fat emulsion containing soybean oil, medium-chain triglycerides, olive oil, and fish oil: A single-center, double-blind randomized study on efficacy and safety in pediatric patients receiving home parenteral nutrition. J. Parenter. Enter. Nutr..

[37] Burrin D.G., Ng K. (2014). Impact of new-generation lipid emulsions on cellular mechanisms of parenteral nutrition-associated liver disease. Adv. Nutr..

[38] Seida J.C., Mager D.R. (2013). Parenteral ω-3 Fatty Acid Lipid Emulsions for Children with Intestinal Failure and Other Conditions. J. Parenter. Enter. Nutr..

[39] Kapoor V., Malviya M.N. (2019). Lipid emulsions for parenterally fed preterm infants. Cochrane Database Syst. Rev..

[40] Kapoor V., Malviya M.N. (2019). Lipid emulsions for parenterally fed term and late preterm infants. Cochrane Database Syst. Rev..

[41] Lilja H.E., Finkel Y. (2011). Prevention and reversal of intestinal failure-associated liver disease in premature infants with short bowel syndrome using intravenous fish oil in combination with omega-6/9 lipid emulsions. J. Pediatr. Surg..

[42] Diamond I.R., Sterescu A. (2009). Changing the Paradigm: Omegaven for the Treatment of Liver Failure in Pediatric Short Bowel Syndrome. J. Pediatr. Gastroenterol. Nutr..

[43] Nandivada P., Baker M.A. (2016). Predictors of failure of fish-oil therapy for intestinal failure–associated liver disease in children. Am. J. Clin. Nutr..

[44] Antonucci A., Fronzoni L. (2008). Chronic intestinal pseudo-obstruction. World J. Gastroenterol..

[45] Mercer D.F., Hobson B.D. (2013). Hepatic Fibrosis Persists and Progresses Despite Biochemical Improvement in Children Treated with Intravenous Fish Oil Emulsion. J. Pediatr. Gastroenterol. Nutr..

[46] Matsumoto C.S., Kaufman S.S. (2014). Hepatic explant pathology of pediatric intestinal transplant recipients previously treated with ω-3 fatty acid lipid emulsion. J. Pediatr..

[47] Nandivada P., Chang M.I. (2015). The Natural History of Cirrhosis from Parenteral Nutrition-Associated Liver Disease After Resolution of Cholestasis with Parenteral Fish Oil Therapy. Ann. Surg..

[48] Khan F.A., Fisher J.G. (2015). Preservation of Biochemical Liver Function with Low-Dose Soy-Based Lipids in Children with Intestinal Failure Associated Liver Disease. J. Pediatr. Gastroenterol. Nutr..

[49] Wang C., Venick R.S. (2019). Long-Term Outcomes in Children with Intestinal Failure-Associated Liver Disease Treated With 6 Months of Intravenous Fish Oil Followed by Resumption of Intravenous Soybean Oil. J. Parenter. Enter. Nutr..

[50] Rumbo C., Martinez M.I. (2017). Utility of Aminotransferase/Platelet Ratio Index to Predict Liver Fibrosis in Intestinal Failure-Associated Liver Disease in Pediatric Patients. J. Parenter. Enter. Nutr..

